# Essential Oil Yield and Composition of the Balkan Endemic *Satureja pilosa* Velen. (Lamiaceae)

**DOI:** 10.3390/molecules25040827

**Published:** 2020-02-13

**Authors:** Ivanka B. Semerdjieva, Valtcho Zheljazkov, Charles L. Cantrell, Tess Astatkie, Abbas Ali

**Affiliations:** 1 Department of Botany and Agrometeorology, Agricultural University, Mendeleev 12, 4000 Plovdiv, Bulgaria; v_semerdjieva@abv.bg; 2 Crop and Soil Science Department, Oregon State University, 3050 SW Campus Way, 109 Crop Science Building, Corvallis, OR 97331, USA; 3 Natural Products Utilization Research Unit, USDA-ARS, University, MS 38677, USA; charles.cantrell@usda.gov; 4 Faculty of Agriculture, Dalhousie University, PO Box 550, Truro, NS B2N 5E3, Canada; astatkie@dal.ca; 5 National Center for Natural Products Research, The University of Mississippi, University, MS 38677, USA; aali@olemiss.edu

**Keywords:** Balkan endemic, savory, monoterpenes, thymol, carvacrol, chemotype, mosquitoes, biting deterrence

## Abstract

*Satureja pilosa* Velen. senso lato is a Balkan endemic plant that is not well characterized and is found on rocky outcrops of limestone base in Stara Planina (the Balkan Mountains) and the Rhodope Mountains. The objective of this study was to assess the variability of essential oil (EO) content and composition of *S. pilosa* collected at 33 locations across the Balkan and Rhodope Mountains in Bulgaria using advanced statistical methods including cluster analysis. The EO content in dried aboveground biomass varied from 0.52% to 2.03%. More than 21 EO constituents were identified, belonging to the groups of monoterpenes and sesquiterpenes. The monoterpenes were the predominant class, comprising 84.8% to 96.1% of the total EO. Thymol and carvacrol were the major constituents of the phenolic monoterpenoids. Thymol varied from 36.6% to 67.1% and carvacrol varied from 52.4% to 93.0% of the total oil. *p*-Cymene also varied widely, from 9.6%–34.0%. There were significant variations between locations and within a location in the EO content and composition. This study identified several chemotypes: (1) thymol and *p*-cymene; (2) thymol, *p*-cymene and *γ*-terpinene; (3) carvacrol and *p*-cymene; (4) carvacrol, *p*-cymene and *γ*-terpinene; and (5) carvacrol. This is the first comprehensive study on the endemic plant *S. pilosa* that identified several chemotypes based on the EO composition. The *S. pilosa* EO from the five different chemotypes exhibited larvicidal and mosquito repellent activity against *Aedes aegypti*. The identified chemotypes can be utilized for the development of new varieties with desirable compositions to meet specific industry needs and new mosquito management control products.

## 1. Introduction

The genus *Satureja* (Lamiaceae) includes about 235 savory species of aromatic plants [1]. They are widespread in the Mediterranean region, Asia, and America [2]. In Europe, there are 12 naturally distributed *Satureja* (savory) species [3], of which 5 species are found in the Bulgarian flora [4]. Savory species and their essential oil (EO) are traditionally used as spice and natural preservatives for food, as well as in the perfume, cosmetic, and pharmaceutical industries [5]. *Satureja* species have broad pharmacological activities, including more than 50 different activities of savory herbage and EO. However, these activities were reported mostly for the annual *S. hortensis* L. (summer savory) and the perennial *S. montana* L. (winter savory) species [6]. Some of the well-known pharmacological activities of savory include stimulation of digestion, antiseptic, anti-inflammatory activity, and for the treatment of premature ejaculation [7,8]. In addition, various activities have been reported such as antioxidant, antibacterial, and antifungal efficacy [9,10,11,12,13,14,15,16,17]; cytotoxic activity [18]; and insecticidal and insect repellent activity [19].

The phytochemical studies on *Satureja* focused mainly on the cultivated species *S. hortensis* and *S. montana*, while little attention was given to the wild *Satureja* species found in natural populations. The phytochemical composition of savory includes a wide range of secondary metabolites such as EO, phenolic acids, and flavonoids [6]. The EO of *Satureja* is characterized with a high percent of oxygenated monoterpenes, among which, the best known EO constituents are thymol, *p*-cymene, and carvacrol [6]. Terpenoids have been suggested as potential taxonomic markers at the genus level [20].

*Satureja pilosa* Vel. (senso lato) is a Bulgarian endemic species according to the official Flora of Europe book [3]. However, there have been recent reports that the species was also found in the flora of Greece and Western Turkey, in regions very close to Bulgaria [21]. Therefore, this species is now considered a Balkan endemic species. In Bulgaria, *S. pilosa* is spread on rocky habitats of limestone base in Stara Planina (Balkan Mountains, central and eastern part), and Rhodope Mountains [4]. Generally, the species has a limited distribution range. The populations of *S. pilosa* are included in the European ecological network EU NATURA 2000 under Directive 92/43/EEC on Habitats, National and Nature Parks, Reserves and Protected Areas. The limited distribution of *S. pilosa* is the main reason why relatively few studies have been conducted on this species. For the territory of Western Turkey, Tümen et al. [21] reported that *S. pilosa* had 2.7% oil content with major constituents being carvacrol (38% to 53%) and *γ*-terpinene (4% to 14%). Dardioti et al. [22] examined 19 populations of *S. pilosa* Velen. subsp. *origanita* and found that 10 populations had EO with major constituent carvacrol (up to 62.3% of total oil) had oregano aroma, 7 populations had *p*-cymene (up to 49.8%) and/or thymol (up to 48.1%) and had thyme aroma, and plants from two other populations had linalool (59.2% and 82.7%) as a major EO constituent and had a lavender-type aroma [22].

One of the first studies of *S. pilosa* in the Bulgarian flora was conducted by Genova and Balinova [23] who reported EO yield varying from 1.68% to 3.11% in four populations, and carvacrol (5.1% to 75%), thymol (0.4% to 51.5%), isomenthone (6.2% to 27.7%), *p*-cymene (12.8% to 15.3%), *γ*-terpinene + dipenten (5.1% to 10%), menthol, isomenthol (1.4% to 2.5%) as its main EO components. Based on these four populations, the cited authors concluded that there were two chemotypes: (1) a thymol type and (2) a carvacrol chemotype [23]. In another study of *S. pilosa* in Bulgaria, Konakchiev and Tsankova [24] reported 1% to 3% EO yield of *S. pilosa* and thymol and carvacrol as the main constituents. However, these two studies were on limited number of populations, the analyses were done using a single sample per population, without replicates, and had no statistical analyses. Generally, research has shown that the genetics, climate, soils, precipitation, time of harvesting, phenological phase, and other factors may have a significant effect on the composition of the EO [16,25,26,27,28].

Overall, most of the published research on the phytochemical composition of *Satureja* lacks statistical data processing; very often, only one plant per population was analyzed without replications, and plants were used from either one or a limited number of populations. These identified research gaps have created difficulties in interpreting the results. Much broader studies are needed to reveal the diversity and variation of the *S. pilosa* EO content and composition and potentially to select plants with a specific composition. In order to establish *S. pilosa* as a new cultivated crop, it is necessary to identify prospective populations with a high content of EO and a desirable composition.

Therefore, the objectives of this study were to establish the diversity and variation of *S. pilosa* EO content and composition in Bulgaria and to identify prospective populations to be used for further selection and breeding using advanced statistical methods. The working hypothesis was that the EO content and composition of *S. pilosa* in different populations across Bulgaria vary significantly, and its EO will have differential bioactivity against mosquitoes.

## 2. Results

### 2.1. Total EO Content (Yield)

Overall, the EO yield of the plants from the 33 locations (populations) varied from 0.52% (Samokitka1) to 2.03% (Maglij/Selci 4) (Table 1).

### 2.2. Qualitative Composition of Essential Oil (EO)

#### 2.2.1. Class Monoterpenes (Phenolic Monoterpenoids, Aromatic Monoterpenes, Monoterpenes Hydrocarbons, Monocyclic Monoterpenes) (Supplementary Table S1)

Monoterpenes was the major chemical class of substances in the composition of *S. pilosa*. In this study, the monoterpenes ranged from 84.8% to 96.1% of the total oil (Table 2). From the monoterpenes class, the predominant subclasses were phenolic monoterpenoids (thymol and carvacrol); hydrocarbon monoterpenes (myrcene, α-terpinene); aromatic monoterpenes (*p*-cymene), and monocyclic monoterpenes (*γ*-terpinene) (Table 1 and Table 2). Thymol (>36.6%) was the main constituent in 20 of the investigated 33 locations, while carvacrol (up to 44.4%) was the main EO constituent in the plants from 12 locations (Table 2). The percent compositions of thymol varied from 0.21% (Taja4, Maglij/Selci5) to 67.1% (Sushica/Karlovo3) (Table 3). Carvacrol ranged from 2.6% to 93.0% (Table 3). The percent composition of *p*-cymene ranged from 9.6% (Kalofer/Panicite1) to 34.0% (Taja 3). *P*-Cymene was absent in the EOs of three locations (Maglij/Selci5, 6; Taja4). The fourth major EO constituent (% by weight) of the monoterpenes class was *γ*-terpinene, and it varied from non-detected in four of the samples to 12.4% (Table 1). The four locations with *p*-cymene under the detection limit were also lacking *γ*-terpinene (Table 1). Myrcene and α-terpinene are components of monoterpenes found in negligible amounts in the tested locations. Myrcene was found in 30 locations, ranging from 0.2% (Maglij/Selci 3) to 2.3% (Samokitka 1). The highest percent composition of α-terpinene was found in Samokitka2 location (3.2%) (Table 1).

#### 2.2.2. Class Sesquiterpenes (Monocyclic Sesquiterpene; Byciclic Sesquiuterpene; Tricyclic Sesquiterpene)

Class Sesquiterpenes comprised 1.4% to 7.96% of the phytochemical composition of S. pilosa (Table 2), with the highest percent compositions being of trans-caryophyllene and caryophyllene oxide. Caryophyllene oxide and trans-caryophyllene ranged from 0.6% to 4.0% and from non-detected to 2.63% (Table 3), respectively.

### 2.3. Anti Mosquito Activity

**Figure 1:**
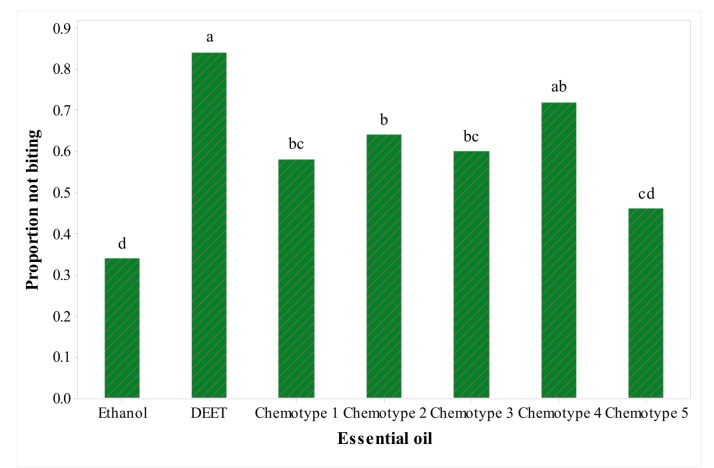
Proportion not biting (PNB) values of essential oils (EOs) from five different chemotypes of *Satureja pilosa* and DEET against *Aedes aegypti*. The EOs were tested at 10 µg/cm^2^ and DEET at 25 nmol/cm^2^ was the positive control. Mean proportions sharing the same letter are not significantly different.

Biting deterrent activity of the essential oils of different chemotypes of *S. pilosa* is presented in Figure 1.

**Figure 2:**
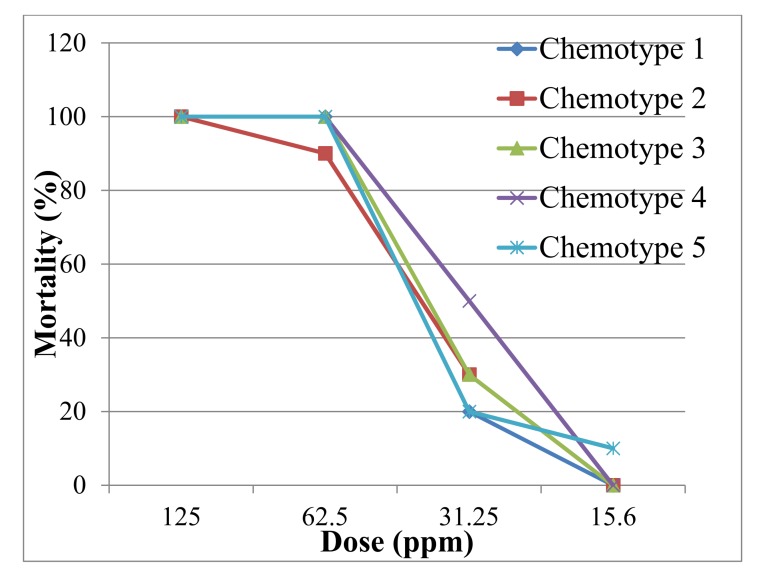
Toxicity of essential oils from different chemotypes of *S. pilosa* against 1-d old larvae of Aedes aegypti.

All the EOs, except chemotype five (carvacrol), showed biting deterrence above the ethanol control. Biting deterrent activity of chemotype four (carvacrol, *p*-cymene and *γ*-terpinene) with proportion not biting (PNB) value of 72 was similar to DEET (Figure 1). All the five EOs representing the five *Satureja* chemotypes tested in this screening bioassay showed larvicidal activity against 1-d old *Aedes aegypti*. The mortality in all the four EOs was similar. At the lowest dose of 15.6 mg/kg, chemotype five gave 10% mortality whereas the other essential oils did not show any mortality (Figure 2).

Four pure compounds, carvacrol, thymol, *ϒ*-terpinene and *p*-cymene, were selected for larvicidal activity. Since the data on carvacrol and thymol has already been reported [29], only *ϒ*-terpinene and *p*-cymene were tested for their larvicidal activity. *ϒ*-Terpinene with LC_50_ value of 27.2 was more active than *p*-cymene (LC_50_ = 36.9) (Table 4).

## 3. Discussion

### 3.1. Essential Oil (EO) Content (Yield)

There was a significant variation in the EO content and composition of S. pilosa samples collected from different locations. The EO yield was around 1% or more in 20 of the locations, which is similar to the one in previous reports [21,23,24]. The EO yields in the remaining 13 locations were between 0.32% and 0.89%. The resulting variations were probably due to a number of factors, including genetic, physiological, ecological, edaphic, and technical, which could not be differentiated here.

### 3.2. Class of Essential Oil (EO) Constituents

**Figure 3:**
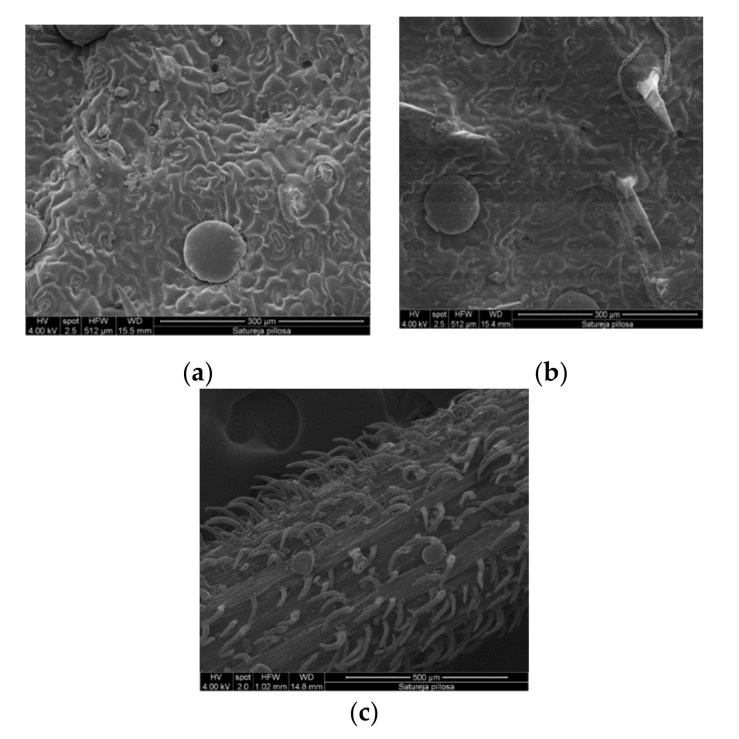
Scanning Electron Microscopy (SEM) analysis of *Satureja pilosa* L. leaf surfaces; adaxial (**a**) and abaxial (**b**) epidermis. Essential oil sessile glands are visible on both the adaxial and abaxial surfaces and also on the petiolate (**c**).

The EO of plants is most often synthesized and accumulated in specialized structures, such as glandular trichomes and idioblasts. These structures may be species-specific, characteristics that allow them to be used as taxonomic traits [30]. In *S. pilosa*, the epidermal trichomes (hairs) are of two types: simple conic and glandular trichomes, and these are the sites for EO accumulation (Figure 3).

A glandular trichome is made of one basal cell and 1–6 cells that form a secretory head. Each glandular trichome is surrounded by 8–16 basic epidermal cells (Figure 3). Essential oil accumulates in the space in the secretory head, the cuticle, and the apical walls of the epidermis. The glandular storage capacity of EO in savory plants is directly related to the formation of glycoside content in their phytochemical composition [31,32]. However, the amount of free thymol and carvacrol, which are stored in the glandular trichomes, is 30 to 400 times higher than that of glycosidically bound forms [31,32].

**Figure 4:**
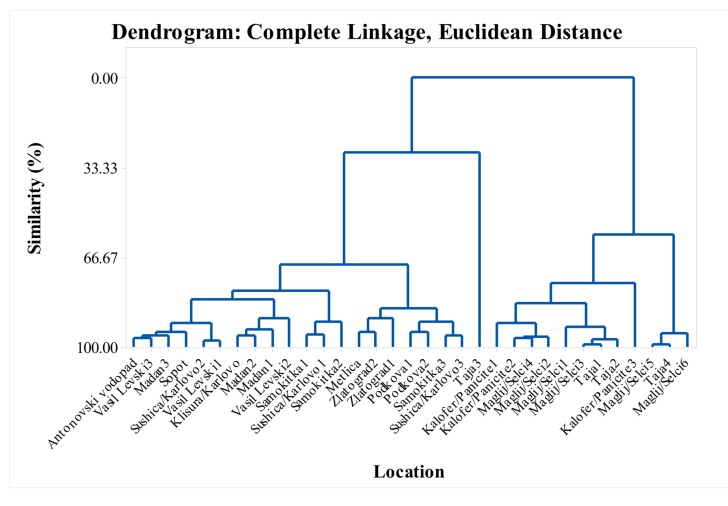
Complete linkage dendrogram showing the similarity of 33 locations in terms of myrcene, α-terpinene, *p*-cymene, *γ*-terpinene, thymol, carvacrol, *trans*-caryophyllene, and caryophyllene oxide of *Satureja pilosa*.

The *S. pilosa* EO composition includes two major classes of compounds; monoterpenes and sesquiterpenes. In this study, although only eight major constituents were statistically analyzed (Figure 4), more than 20 EO constituents were found and identified in the *S. pilosa* EO, some being reported for the first time (oct-1-en-3-ol, carvacrol methyl ether, cis-*β*-ocimene, *p*-cymen-8-ol; endo-borneol; *trans*-caryophyllene) (Supplementary Table S1).

**Figure 5:**
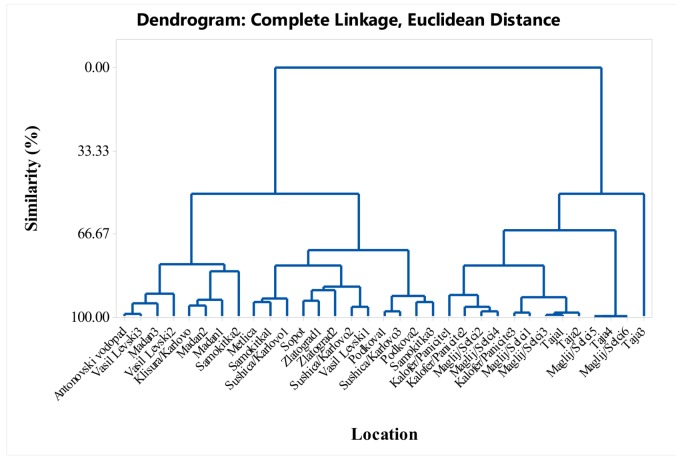
Complete linkage dendrogram showing the similarity of 33 locations in terms of thymol and *p*-cymene of *Satureja pilosa*.

**Figure 6:**
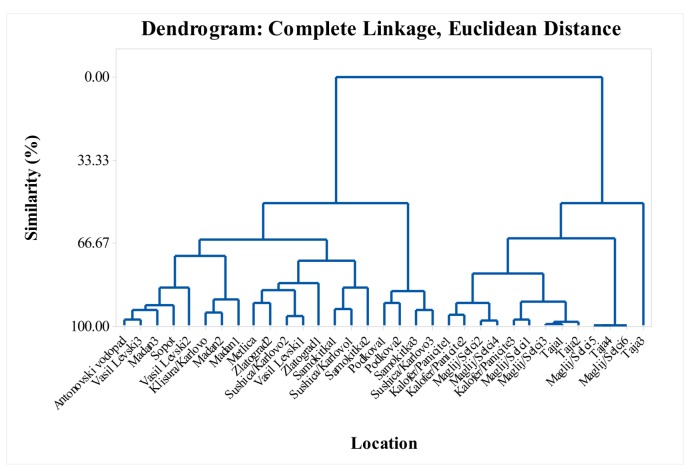
Complete linkage dendrogram showing the similarity of 33 locations in terms of thymol, *p*-cymene, and *γ*-terpinene of *Satureja pilosa*.

**Figure 7:**
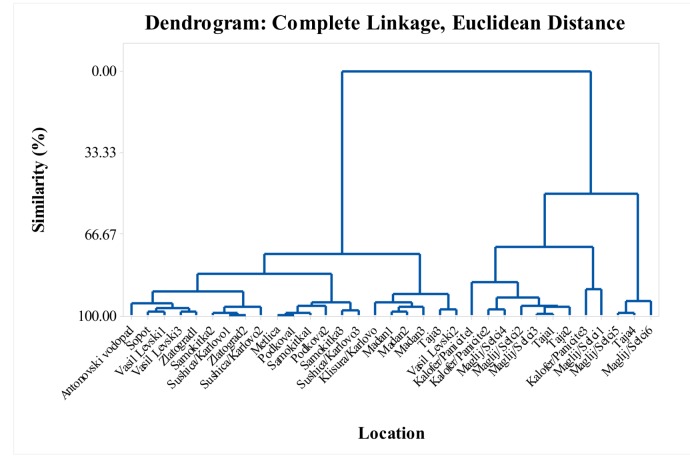
Complete linkage dendrogram showing the similarity of 33 locations in terms of carvacrol and *p*-cymene of *Satureja pilosa*.

**Figure 8:**
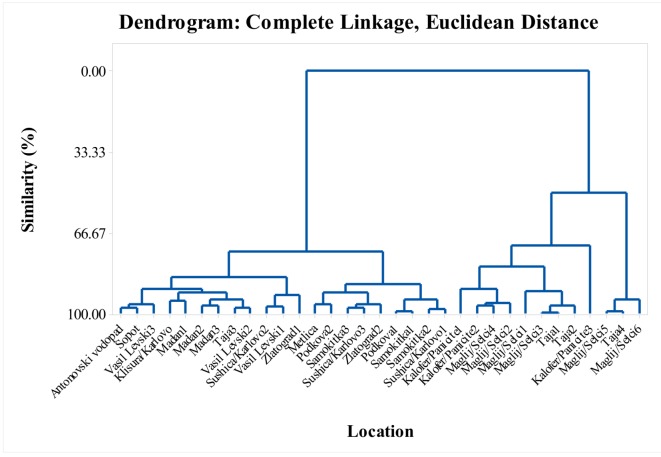
Complete linkage dendrogram showing the similarity of 33 locations in terms of carvacrol, *p*-cymene, and *γ*-terpinene of *Satureja pilosa*.

In this study, the monoterpenes were the dominant class of compounds, and reached 96.1% of the total EO composition in some locations. Six major constituents predominated in the monoterpenes class; thymol, carvacrol, myrcene, α-terpinene, *p*-cymene, and *γ*-terpinene (Figure S1). In this *S. pilosa* study, these main components of the monoterpenes were predominant in different percentage ratios. Depending on % ratio of the major constituents of the monoterpenes, the studied *S. pilosa* samples can be subclassified as follows: (1) thymol and *p*-cymene (Figure 5), (2) thymol, *p*-cymene and *γ*-terpinene (Figure 6), (3) carvacrol and *p*-cymene 9 (Figure 7), (4) carvacrol, *p*-cymene and *γ*-terpinene (Figure 8), and (5) carvacrol. The lowest values (lowest percent composition limits) for the respective constituents in the above groups were: 36.6% for thymol, 13.6% for *p*-cymene, 2.68% for *γ*-terpinene; and 52.4% for carvacrol. Thymol, *p*-cymene, and carvacrol are characteristic EO constituents in savory species [6], and these constituents have been shown to have a common biosynthetic pathway. Based on the radioactively labeled monoterpenes experiments with thyme, Poulose and Croteau [33,34] demonstrated that the biosynthesis of thymol starts with *γ*-terpinene as initial monoterpene substrate and proceeds via the aromatic *p*-cymene as an intermediate. The authors proposed that the occurrence of *γ*-terpinene and *p*-cymene with either one or both phenolic monoterpenes suggests a common mechanism for thymol and carvacrol biosynthesis.

Dardioti et al. [22] reported that “oregano” aroma of *Satureja* plants is due to the fact that carvacrol is its main EO constituent, and the “thyme” aroma of other *Satureja* plants was due to thymol being their major EO constituent. However, Dardioti et al. [22] found two populations with a linalool-rich oil (59.2% and 82.7%) and a prominent “lavender” aroma that were not found in this study. In this study, we cannot confirm such aromas, especially for the “lavender” one.

Moreover, the phytochemical data on *S. pilosa* in this study was substantially different from the one reported by Genova and Balinova [23] in four populations of *S. pilosa* in Bulgaria. In addition to carvacrol and thymol, the authors cited the presence of isomenthone (6.2% to 27.7%), dipenten (5.1% to 10%), menthol, isomenthol (1.4% to 2.5%) [23], constituents not found in any EO from this study. Dissimilar EO composition of *S. pilosa* was also reported by Konakchiev and Tsankova [24]. In the sample (from one population only), they found the traditional thymol and *p*-cymene but also constituents not found in this study (such as α-phellandrene, δ-3-carene, limonene, terpinolene, *trans*-sabinene hydrate, α-copaene, *p*-bourbonene, β-gurjunene, α-humulene, germacrene D) [24].

It is evident from literature review and the results from this study that the *S. pilosa* EO composition is quite variable. In this study, samples collected from the same geographic populations showed a significant variation in EO composition (Figure 4). Of the 33 samples analyzed, only 4 samples were collected as one sample per location site; three to six samples per location were collected from all other locations. From the results in Table 2 and Table 3, it can be seen that within the same habitat, the derived values of the EO constituents for the individual samples in a given location were variable. For example, in some samples collected at Maglij/Selci (1, 2, 3, 4, 5, and 6) the EO contained all the main constituents of the monoterpene class. In some other samples of the same population, carvacrol (88.4% to 93.0%) was the main constituent of the EO while the remaining monoterpenes were absent (Table 3).

The dendrogram in Figure 4 supports the variability shown in the tables. The similarity of the locations in the dendrogram in terms of their myrcene, α-terpinene, *p*-cymene, *γ*-terpinene, thymol, carvacrol, *trans*-caryophyllene, and caryophyllene oxide content formed six cluster groups with over 85% similarity (Figure 4). Cluster groups include samples collected from various locations in the Balkan Mountains and the Eastern Rhodope Mountains, which are characterized by different climatic conditions. As separate groups are samples from Karlovo, Kalofer, Taja, and Seltsi, which demonstrates the significant variability in the composition of the EO even within one floristic region (Figure 5, Figure 6, Figure 7 and Figure 8). The savory populations from the Karlovo, Kalofer, Taja and Selci locations are situated only 10–15 km apart and therefore, all these could be considered as one geographic population. The plants at these locations grew on similar rocky habitats, on a limestone base in the Balkan Mountains, under the influence of temperate continental climate. The chemical composition of some samples of *S. pilosa* collected from the Eastern Rhodope Mountains were similar to those of the samples collected in the Balkan Mountains (Figure 4). The climate in the Eastern Rhodopes is characterized as transition to Mediterranean climate, because the altitude is lower, and the river valleys facilitate the movement of Mediterranean air from the south to the north [35].

In this study, the observed dynamics of the main monoterpenes’ thymol and carvacrol in *S. pilosa* did not generate significant correlation or any trend between the chemical composition and the climatic characteristics of the populations. The cluster groups in Figure 5, Figure 6, Figure 7 and Figure 8 combine samples from both the Balkan Mountains and the Eastern Rhodope Mountains. High percent compositions of thymol and carvacrol were found both in locations under continental climate and under Mediterranean climate. Therefore, the findings of this study contradict the conclusions of Dardioti et al. [22] for *Satureja pilosa* subsp. *origanita*, who reported that the EO content and the amount of carvacrol gradually decreased from the Meso-mediterranean to the Temperate Axeric bioclimates, while the amounts of *p*-cymene, thymol, and linalool increased [22].

Genotype and developmental stages may play a major role in the phytochemical composition of *S. pilosa*. Overall, the results in this study support the hypothesis that the *S. pilosa* EO at different locations and within a location may be variable and have different qualitative and quantitative composition. In this study, *S. pilosa* EO yield and composition varied at both interpopulation and intra-population levels.

The next class of compounds in the *S. pilosa* EO composition were sesquiterpenes (tricyclic sesquiterpene). Overall, this class of compounds was in insignificant amounts and in similar percent composition ranges between samples.

### 3.3. Anti Mosquito Activity

Mosquitoes are economically important pests because they serve as vectors for the transmission of pathogens and parasites [36]. The development of environmentally friendly and effective mosquito control products is a challenge for researchers for several reasons: (1) mosquitoes develop resistance to the pesticides used, (2) the need for environmental protection, and (3) protecting human health. The potential negative effects of synthetic insecticides have led researchers to look into new alternatives that would be acceptable from environmental and public health perspectives [37]. The use of plant-based insecticides is an alternative for mosquito protection [36,37]. The Lamiaceae plant family is rich in species that accumulate natural products with potential as mosquito control agents [38,39]. The results from this study for *S. pilosa* anti mosquito activity are being reported for the first time. Indeed, the anti mosquito effect of *Satureja* EO is logical because of the presence of carvacrol, thymol, *p*-cymene and *γ*-terpinene in these oils. These EO constituents are known to be effective repellent chemicals [40]. The toxicity of two major EO constituents, carvacrol and thymol, with LC_50_ values of 13.9 and 20.1 mg/kg, respectively, were previously reported by Tabanca et al. [29].

## 4. Materials and Methods

### 4.1. Materials

The materials used in this study were randomly selected aboveground plant parts of *Satureja pilosa* Velen. (senso lato) from each of the 33 collection locations (Table 5). The samples of the species were collected at the end of July and the beginning of August, during mass flowering stage. Voucher specimens of *Satureja pilosa* were deposited at the Herbarium of the Agricultural University, Plovdiv, Bulgaria (SOA) [41]. The 33 collection locations of *S. pilosa* with the exact coordinates and altitude are presented in Table 5. Prior to the EO isolation, all *Satureja* biomass samples were air dried for approximately 30 days in a shady area below 35 °C temperatures to minimize oil losses and changes in the EO profile.

Subsamples were generated randomly from each air-dried sample that included all aboveground plant parts of *S. pilosa*. The EO was extracted via hydrodistillation in 2-L distillation Clevenger units (Laborbio Ltd. Sofia, Bulgaria, laborbio.com) following the method description of the British Pharmacopeia [42]. The EO extraction was done at the Research Institute for Roses and Medicinal Plants in Kazanluk, Bulgaria, and each extraction was performed in two replicates. Samples of dried aboveground plants (Table 5) plus 0.8 L of water were placed in the Clevenger apparatus for oil separation during distillation. After isolation of each subsample, EO volume and weight were measured, and the EO samples were stored in a freezer at −4 °C until the analyses. This study reports the oil content (yield) based on weight in air dried biomass. Commercial standards for myrcene, α-terpinene, *p*-cymene, *γ*-terpinene, thymol, carvacrol, *trans*-caryophyllene, and caryophyllene oxide were obtained from Sigma-Aldrich (St. Louis, MO, USA).

### 4.2. Gas Chromatography (GC), Mass Spectroscopy (MS) Methods and Analyses of the EO

The isolated EO from all *Satureja* samples in two replications were analyzed for chemical profile by gas chromatography (GC)–mass spectroscopy (MS)–flame ionization detection (FID) techniques. Using a micropipette, 50 μL of oil (weight measured on a tared balance) from each sample was transferred into a 10 mL volumetric flask. Samples were brought to volume with CHCl_3_. A 1 mL aliquot of each diluted oil sample was placed by glass pipet into a GC vial for analysis. Oil samples were analyzed by GC-MS-FID on an Agilent 7890A GC system equipped with an Agilent 5975C inert XL MSD with triple axis detector and an Agilent 7693 autosampler. Chemical standards and oils were analyzed using a DB-5 column (30 m × 0.25 mm fused silica cap. column, film thickness of 0.25 µm) operated using the following conditions: injector temp., 240 °C; column temp., 60 to 240 °C at 3 °C/min, held at 240 °C for 5 min; carrier gas, He; injection volume, 1 µL (split ratio 25:1); MS mass range from 50 to 550 m/z; filament delay of 3.5 min; injection volume, 1 μL (split ratio 50:1); FID temperature was 300 °C. Post-column splitting was performed so that 50% of outlet flow proceeds to FID and 50% to mass spectrometry (MS) detection. All but two compounds were identified by Kovat Index analysis [43], direct comparison of MS and retention time to authentic standards and comparison of mass spectra with those reported in the NIST mass spectra database. α-thujene and thymohydroquinone were identified by only Kovat Index analysis and comparison of mass spectra with those reported in the NIST mass spectra database since commercial standards were not available. For all other compounds, commercial standards were purchased from Sigma-Aldrich (St. Louis, MO, USA). Standards were injected and compared with retention time and mass spectra data of oil and used for identification. Compounds were quantified by performing area percentage calculations based on the total combined FID area. For example, the area for each reported peak was divided by total integrated area from the FID chromatogram from all reported peaks and multiplied by 100 to arrive at a percentage. The percentage of a peak is a percentage relative to all other constituents integrated in the FID chromatogram.

### 4.3. Scanning Electron Microscopy (SEM) Analysis of Leaves

The scanning electron microscope (SEM) used in this investigation was an FEI Quanta 600 SEM at the Microscopy Facility at Oregon State University, United States. Sample preparation included placing small samples into a fixative, 1% paraformaldehyde and 2.5% glutaraldehyde in 0.1M sodium cacodylate buffer with pH 7.4. The samples were soaked in fixative for 2 h, followed by two rinses in 0.1M cacodyalte buffer, 15 min each, and dehydration in acetone (10%, 30%, 50%, 70%, 90%, 95%, 100%), 10–15 min each, followed by critical point drying (two ‘bomb flushes’ at chamber pressure to 5 °C, fill chamber with CO_2_). The samples were left to vent for five min, and then, the procedure was repeated. The dry samples were mounted onto an aluminum SEM stub with double stick carbon tape. Samples were sputter coated with a Cressington 108A sputter coater from Ted Pella with Au/Pd, 60/40 mix. For leaf surfaces, the terminology and classification by Barthlott and Ehler [30] were used.

### 4.4. Anti Mosquito Activity

#### 4.4.1. Insects

Yellow fever mosquitoes used in these studies were from a laboratory colony maintained at the Mosquito and Fly Research Unit, Center for Medical, Agricultural and Veterinary Entomology, USDA-ARS, Gainesville, Florida since 1952. Mosquitoes were reared to the adult stage by feeding the larvae on a larval diet of 2% slurry of 3:2 Beef Liver powder (now Foods, Bloomingdale, Illinois) and Brewer’s yeast (Lewis Laboratories Ltd., Westport, CT). The eggs were hatched and the larvae reared to pupal stage in an environment controlled room at a temperature maintained at 27 °C ± 2 °C and 60 ± 10% RH in a photoperiod regimen of 12:12 (L:D) h. The adult mosquitoes were maintained in laboratory using the procedures described by Ali et al. [44].

#### 4.4.2. In Vitro Klun and Debboun (K&D) Bioassay

Bioassays were conducted using a six-celled in vitro Klun and Debboun (K&D) bioassay system [45]. Briefly, the bioassay system consists of six 3 × 4 cm wells each of them containing approximately 6 μL of the feeding solution. As described by Ali et al. [46], a feeding solution consisting of CPDA-1 and ATP was used instead of blood. All the compounds were tested in this study and DEET, 97%, *N, N*-diethyl-meta-toluamide (Cas # 134-62-3, Sigma-Aldrich, St. Louis, MO, USA) at 25 nmol/cm^2^ was used as a positive control. All the treatments were freshly prepared in molecular biology grade 100% ethanol. The temperature of the feeding solution in the reservoirs was maintained at 37 °C by using a circulatory bath. The reservoirs were covered with a layer of collagen membrane (Devro, Sandy Run, SC, USA). The test samples were randomly applied to six 4 × 5 cm marked areas of organdy and positioned over the membrane-covered CPDA-1+ATP solution with a Teflon separator placed between the treated organdy and the module to prevent the contamination. The K&D module containing five female mosquitoes per cell was positioned over treated organdy, and trap doors were opened to expose the treatments to the females. The number of mosquitoes biting through treated organdy in each cell was recorded after a 3 min exposure, and mosquitoes were prodded back into the cells to check the actual feeding. These mosquitoes were then squashed to determine the numbers that had imbibed the solution. A replicate consisted of six treatments: four test samples, DEET, and ethanol treated organdy as solvent control. Two sets of five replications each with five females per treatment were conducted on two different days using a newly treated organdy and a new batch of females in each replication. Treatments were replicated ten times.

#### 4.4.3. Larvicidal Bioassays

Bioassays were conducted using the bioassay system described by Pridgeon et al. [47]. Further methods were described in Ali et al. [44]. Dimethyl sulfoxide (DMSO) was used as a solvent to prepare the treatments and was also used as a negative control. Permethrin (95.7%) (Chem Service, Inc. West Chester, PA, USA) was used as a positive control.

### 4.5. Statistical Analyses

Analysis of Variance (ANOVA) of a Completely Randomized Design (CRD) with two replications was conducted to determine the effect of location (33 levels for *Satureja pilosa*) on essential oil (EO) yield, and the percent compositions of eight constituents (myrcene, α-terpinene, p-cymene, γ-terpinene, thymol, carvacrol, trans-caryophyllene, and caryophyllene oxide). The effect of chemotype (seven levels) on Proportion not biting (PNB) values in K&D calculated using the following formula:

(1) $$PNB=1-\frac{Total\;Number\;of\;Females\;Biting}{Total\;Number\;of\;Females}$$

The analyses were completed using the Mixed Procedure of SAS [48]. Since the effect of Location on EO yield and the percent compositions of all constituents and the effect of Chemotype on PNB were significant (*p* value < 0.05), further multiple means comparison was completed using Tukey’s multiple range test at 5% level of significance, and letter groupings were generated. LC_50_ values for larvicidal data were calculated by using SAS, Proc Probit. For each response variable, the validity of model assumptions was verified by examining the residuals as described in Montgomery [49], and appropriate transformations were applied on response variables with violated assumptions. The results reported in the tables are back transformed to the original scale. To determine the similarity level of the 33 locations in terms of (1) all 8 constituents; (2) thymol and *p*-cymene; (3) thymol, *p*-cymene, and *γ*-terpinene; (4) carvacrol and *p*-cymene; and (5) carvacrol, *p*-cymene, and *γ*-terpinene multivariate analysis of complete linkage clustering [50] was conducted, and dendrograms were produced.

## 5. Conclusions

This study assessed the variability of essential oil (EO) content and composition of the Balkan endemic plant *S. pilosa* collected at 33 locations across the Balkan and Rhodope Mountains in Bulgaria. The EO content in dried aboveground biomass varied significantly, from 0.52% to 2.03%. Overall, 22 EO constituents were identified with monoterpenes being the predominant class. Thymol (36.6% to 67.1%), carvacrol (52.4% to 93.0%), and *p*-cymene (9.6% to 34.0%) were the major oil constituents. This study identified five chemotypes, based on the % ratio of the major constituents of the monoterpenes: (1) thymol and *p*-cymene; (2) thymol, *p*-cymene and *γ*-terpinene; (3) carvacrol and *p*-cymene; (4) carvacrol, *p*-cymene and *γ*-terpinene; and (5) carvacrol. Further research and more detailed intrapopulation studies may be needed in order to reveal the diversity of the EO composition of the species. The *S. pilosa* EO has shown anti mosquito activity against *Aedes aegypti*.

## Figures and Tables

**Table 1:** Mean EO yield (%) and the percent compositions (%) of myrcene, α-terpinene, p-cymene, and γ-terpinene (major *Satureja pilosa* constituents) collected at 33 locations.

Location	EO Yield	Myrcene	α-Terpinene	*p*-Cymene	*γ*-Terpinene
Antonovski vodopad	0.64 cde	0.48 defg	1.06 abcde	25.87 abcdef	0.65 cdefg
Kalofer/Panicite1	1.16 abcde	1.11 abcdef	0.76 cde	9.63 g	6.88 abc
Kalofer/Panicite2	0.99 abcde	1.21 abcde	0.97 abcde	12.79 fg	7.55 ab
Kalofer/Panicite3	0.73 abcde	0.73 bcdefg	0.80 cde	23.96 abcdef	3.75 abcde
Klisura/Karlovo	0.80 abcde	0.87 abcdefg	1.62 abcde	28.36 abcde	3.15 abcde
Madan1	0.76 abcde	1.43 abcd	2.85 ab	29.71 abc	6.65 abc
Madan2	1.44 abcd	0.79 abcdefg	1.28 abcde	30.99 ab	0.90 bcdef
Madan3	1.22 abcd	0.27 fg	1.02 abcde	28.65 abcd	0.06 fg
Maglij/Selci1	1.62 abc	0.71 bcdefg	0.956 abcde	22.70 abcdef	2.35 abcdef
Maglij/Selci2	1.69 abc	0.93 abcdef	1.05 abcde	15.63 bcdefg	4.89 abcd
Maglij/Selci3	1.04 abcde	0.20 g	0.48 e	18.50 abcdefg	0.16 defg
Maglij/Selci4	2.03 a	0.96 abcdef	0.89 bcde	14.42 cdefg	5.15 abcd
Maglij/Selci5	1.74 abc	0.00 h	0.00 f	0.00 h	0.00 g
Maglij/Selci6	1.80 abc	0.00 h	0.00 f	0.00 h	0.00 g
Metlica	0.75 abcde	1.47 abcd	1.89 abcde	13.59 efg	6.71 abc
Podkova1	1.10 abcde	2.03 ab	2.56 abc	13.31 fg	11.16 a
Podkova2	1.31 abcd	1.57 abcd	1.82 abcde	10.10 g	6.55 abc
Samokitka1	0.52 de	2.26 a	2.89 ab	12.46 fg	11.96 a
Samokitka2	1.70 abc	2.08 ab	3.19 a	18.30 abcdefg	12.43 a
Samokitka3	0.66 bcde	0.96 abcdef	1.39 abcde	14.02 defg	3.24 abcde
Sopot	0.89 abcde	0.31 efg	0.48 e	24.18 abcdef	0.19 efg
Sushica/Karlovo1	1.92 ab	1.53 abcd	2.28 abcd	17.24 abcdefg	11.01 a
Sushica/Karlovo2	0.34 e	1.24 abcde	1.77 abcde	20.02 abcdefg	5.32 abcd
Sushica/Karlovo3	1.00 abcde	0.46 defg	0.71 cde	14.64 cdefg	1.77 abcdef
Taja1	1.37 abcd	0.20 g	0.67 de	18.51 abcdefg	0.00 g
Taja2	0.98 abcde	0.28 fg	0.54 e	19.33 abcdefg	0.06 fg
Taja3	1.12 abcde	0.60 cdefg	1.19 abcde	34.03 a	1.94 abcdef
Taja4	1.20 abcde	0.00 h	0.00 f	0.00 h	0.00 g
Vasil Levski1	0.64 cde	1.07 abcdef	1.55 abcde	22.71 abcdef	4.55 abcd
Vasil Levski2	0.89 abcde	0.60 cdefg	1.34 abcde	31.62 ab	2.65 abcdef
Vasil Levski3	1.20 abcde	0.70 bcdefg	1.99 abcde	25.41 abcdef	2.30 abcdef
Zlatograd1	0.80 abcde	1.81 abc	2.40 abcd	24.01 abcdef	11.09 a
Zlatograd2	0.68 bcde	1.51 abcd	1.29 abcde	17.49 abcdefg	2.68 abcdef

^1^ Within each column the means that share the same letter are not significantly different.

**Table 2:** The class compounds in *Satureja pilosa* in different locations.

Location	Compound				Location	Compound			
	Monoterpenes (%)	Sesquiterpenes (%)	Other (%)	Unknown (%)		Monoterpenes (%)	Sesquiterpenes (%)	Other (%)	Unknown (%)
1. V.Levski1	89.3	2.16	5.22	0.94	18.Antonovski vodopad	84.8	2.68	5.71	0.78
2. V.Levski2	90.5	4.18	3.79	1.23	19.Klisura/Karlovo	92.9	4.06	3.1	1.10
3. V.Levski3	90.5	2.93	6.29	0.78	20. Sopot	87.3	5.76	5.48	1.60
4. SushicaKarlovo2	92.7	3.89	1.6	1.40	21.Maglij/Selci2	94.2	3.53	1.16	1.34
5. SushicaKarlovo3	91.8	2.5	4.89	1.18	22.Maglij/Selci1	92.7	3.78	1.98	1.59
6. SushicaKarlovo1	87.2	4.3	5.81	1.45	23.Maglij/Selci3	92.9	3.88	0.97	1.54
7. Podkova1	93.4	3.20	1.06	2.09	24.Maglij/Selci4	95.2	2.3	1.39	1.23
8. Podkova2	92.2	3.66	1.28	1.21	25.Maglij/Selci 5	89.6	6.4	2.69	0.22
9. Metlica	93.3	4.53	0.96	1.29	26.Maglij/Selci6	94.2	3.75	1.73	0.26
10. Samokitka2	92.8	3.95	1.83	1.65	27. Taja4	88.9	7.96	2.79	0.31
11. Samokitka1	93.8	2.57	2.48	1.61	28. Taja1	93.0	3.74	2.17	1.2
12.Samokitka3	93.4	3.94	1.38	1.65	29. Taja2	91.5	4.2	3.33	1.33
13. Zlatograd 2	92.7	2.56	2.53	2.08	30. Taja3	93.7	3.98	3.47	0.93
14. Zlatograd 1	93.1	4.19	1.73	1.71	31. Panicite 1	96.1	2.2	0.59	1.11
15. Madan1	91.9	3.55	3.05	1.55	32. Panicite 2	96.0	1.5	0.82	1.43
16. Madan2	89.8	5.65	3.28	1.31	33. Panicite 4	86.5	4.67	7.28	2.12
17. Madan	93.0	2.88	3.13	1.13					

**Table 3:** Percent composition of thymol, carvacrol, *trans*-caryophyllene, and caryophyllene oxide (major *Satureja pilosa* constituents) collected at 33 locations.

Location	Thymol	Carvacrol	*Trans*-Caryophyllene	Caryophyllene Oxide
Antonovski vodopad	49.39 a	2.55 j	1.44 abc	1.83 cd
Kalofer/Panicite1	0.32 c	74.99 abc	1.58 abc	0.59 d
Kalofer/Panicite2	0.35 c	68.82 abcd	0.73 c	0.65 d
Kalofer/Panicite3	0.19 c	52.43 de	1.82 abc	1.94 cd
Klisura/Karlovo	39.51 a	11.17 f	2.64 a	1.77 cd
Madan1	36.61 a	7.65 fghi	0.63 c	0.62 d
Madan2	41.24 a	8.41 fg	0.88 abc	0.89 d
Madan3	50.98 a	5.75 ghij	0.86 abc	1.47 cd
Maglij/Selci1	0.25 c	62.59 cde	2.63 a	1.15 d
Maglij/Selci2	0.95 bc	67.21 abcd	2.03 abc	0.78 d
Maglij/Selci3	0.28 c	69.43 abcd	1.35 abc	2.14 bcd
Maglij/Selci4	0.28 c	70.98 abcd	1.37 abc	0.91 d
Maglij/Selci5	0.21 c	88.36 ab	0.00 d	3.27 abc
Maglij/Selci6	0.30 c	93.04 a	0.00 d	1.88 cd
Metlica	54.09 a	7.43 fghi	2.65 a	1.27 d
Podkova1	66.20 a	7.70 fgh	1.58 abc	0.74 d
Podkova2	63.45 a	6.29 fghi	2.68 a	0.97 d
Samokitka1	50.30 a	7.77 fgh	0.75 bc	0.92 d
Samokitka2	41.60 a	7.11 fghi	1.64 abc	1.24 d
Samokitka3	63.07 a	5.54 ghij	0.70 c	0.74 d
Sopot	54.12 a	4.57 ghij	1.18 abc	3.85 ab
Sushica/Karlovo1	50.37 a	6.53 fghi	2.52 a	1.37 d
Sushica/Karlovo2	51.14 a	4.62 ghij	2.48 ab	1.82 cd
Sushica/Karlovo3	67.09 a	3.63 ij	1.10 abc	1.36 d
Taja1	0.90 bc	69.91 abcd	1.77 abc	1.36 d
Taja2	0.26 c	66.59 bcd	1.75 abc	2.11 bcd
Taja3	5.04 b	44.42 e	2.20 abc	1.77 cd
Taja4	0.21 c	87.42 abc	0.00 d	4.04 a
Vasil Levski1	50.52 a	4.94 ghij	1.62 abc	1.53 cd
Vasil Levski2	47.00 a	3.95 hij	2.13 abc	2.04 bcd
Vasil Levski3	48.82 a	5.87 fghi	1.79 abc	1.78 cd
Zlatograd1	58.44 a	7.02 fghi	1.52 abc	1.09 d
Zlatograd2	56.83 a	6.32 fghi	0.66 c	0.85 d

^1^ Within each column, means sharing the same letter are not significantly different.

**Table 4:** Toxicity of pure compounds from *S. pilosa* chemotypes against 1-d-old larvae of *Aedes aegypti* at 24 h post treatment.

Compound	LC_50_ ppm (95% CI)*^a^*	LC_90_ ppm (95% CI)	χ^2^	df^b^
*p*-cymene	36.9 (33.5–40.7)	54.4 (48.8–64.8)	55	48
*ϒ*-terpinene	27.2 (24.1–30.9)	52.4 (44.4–66.2)	80.2	48

(a) 95% CI is the confidence interval. (b) df refers to degree of freedom.

**Table 5:** Location coordinates, altitude (masl), and sample size (g) of *Satureja pilosa* in Bulgaria.

№	Coordinate	Masl	Location Name	Sample (g)	№	Coordinate	Masl	Location Name	Sample (g)
1	N42°43′54.0″ E024°19′24.5″	1039	Antonovski vodopad	30	18	N41°24′29.6″ E025°26′15.0″	612	Samokitka1	15
2	N42°40′09.05″ E024°59′45.4″	888	Kalofer/Panicite1	20	19	N41°24′29.3″ E025°26′15.3″	612	Samokitka2	35
3	N42°40′10.8″ E024°59′51.1″	935	Kalofer/Panicite2	20	20	N41°24′29.3″ E025°26′15.8″	612	Samokitka3	40
4	N42°40′10.8″ E024°59′51.1″	935	Kalofer/Panicite3	25	21	N42°40′07.0″ E024°44′46.3″	652	Sopot	45
5	N42°42′14.9″ E024°24′34.1″	984	Klisura/Karlovo	30	22	N42°38′39.9″ E024°50′52.4″	666	Sushica/Karlovo1	50
6	N41°32′21.8″ E024°55′06.2″	666	Madan1	45	23	N42°38′42.2″ E024°50′20.8″	666	Sushica/Karlovo2	20
7	N41°32′21.8″ E024°55′06.2″	666	Madan2	25	24	N42°38′44.8″ E024°50′18.0″	669	Sushica/Karlovo3	50
8	N41°32′21.8″ E024°55′06.2″	666	Madan3	45	25	N42°40′02.7″ E025°04′35.2″	663	Taja1	50
9	N42°37′45.3″ E025°33′27.2″	704	Maglij/Selci1	15	26	N42°40′03.2″ E025°04′34.9″	669	Taja2	50
10	N42°37′38.4″ E025°33′27.8″	708	Maglij/Selci2	25	27	N42°40′03.5″ E025°04′35.4″	669	Taja3	50
11	N42°37′49.4″ E025°33′18.9″	731	Maglij/Selci3	35	28	N42°40′02.7″ E025°04′35.3″	662	Taja4	20
12	N42°37′52.2″ E025°33′14.5″	749	Maglij/Selci4	15	29	N42°37′46.7″ E024°54′31.3″	554	Vasil Levski1	45
13	N42°37′52.4″ E025°33′14.5″	749	Maglij/Selci5	25	30	N42°37′46.8″ E024°54′31.3″	554	Vasil Levski1	45
14	N42°37′52.6″ E025°33′14.5″	749	Maglij/Selci6	50	31	N42°37′47.9″ E024°54′26.4″	550	Vasil Levski2	50
15	N41°25′52.2″ E025°26′07.5″	503	Metlica	50	32	N41°23′15.3″ E025°09′57.4″	394	Zlatograd1	25
16	N41°27′53.0″ E025°25′01.5″	562	Podkova1	50	33	N41°23′15.3″ E025°09′57.4″	394	Zlatograd2	15
17	N41°25′50.0″ E025°25′32.9″	511	Podkova2	50					

## Supplementary Materials

The following are available online. Figure S1: Representative chromatogram of *Satureja pilosa* Velen. Table S1: Percent composition range (in %) of the essential oil constituents that were identified in all collected samples of *Satureja pilosa*.
