# Supplementary material for: Essential Oil Yield and Composition of the Balkan Endemic Satureja pilosa Velen. (Lamiaceae)
Source: Molecules. 2020 Feb 13;25(4):827. doi: 10.3390/molecules25040827 (PMC7070593; doi:10.3390/molecules25040827)
Supplement: Supplementary file 1 [file molecules-25-00827-s001.pdf]

Supplemental Table 1. Concentration range (in %) of the essential oil constituents that were identified in all collected samples of *Satureja pilosa*.

Constituent Number	Volatile Constituent	RTime	Concentration Range (min-max, in %)
1	α-thujene	5.843	0.0 – 1.65
2	α-pinene	6.045	0.0 – 1.25
3	camphene	6.454	0.0 – 0.99
4	oct-1-en-3-ol	7.262	0.0 – 1.15
5	myrcene	7.629	0.0 – 2.99
6	α-terpinene	8.514	0.0 – 3.91
7	*p*-cymene	8.813	0.0 – 35.44
8	unknown	8.944	0.0 – 1.46
9	*cis*-β-ocimene	9.205	0.0 – 3.35
10	*γ*-terpinene	10.017	0.0 – 17.21
11	unknown	10.337	0.0 – 0.91
12	unknown	11.530	0.18 - 1.88
13	*endo*-borneol	14.302	0.5 – 4.09
14	terpinen-4-ol	14.755	0.32 – 1.37
15	*p*-cymen-8-ol	15.089	0.07 – 0.72
16	carvacrol methyl ether	17.538	0.00 – 3.93
17	thymoquinone	17.760	0.00 – 9.74
18	thymol	19.958	0.2 – 75.05
19	carvacrol	20.303	2.28 – 93.37
20	trans- caryophyllene	24.986	0.00 – 3.75
21	*β*-bisabolene	28.570	0.00 – 3.49
22	*δ*-cadinene	29.113	0.00 – 1.28
23	spathulenol	31.234	0.00 – 5.76
24	caryophyllene oxide	31.445	0.36 – 4.55
**25**	thymohydroquinone	30,897	0.00 – 1.9

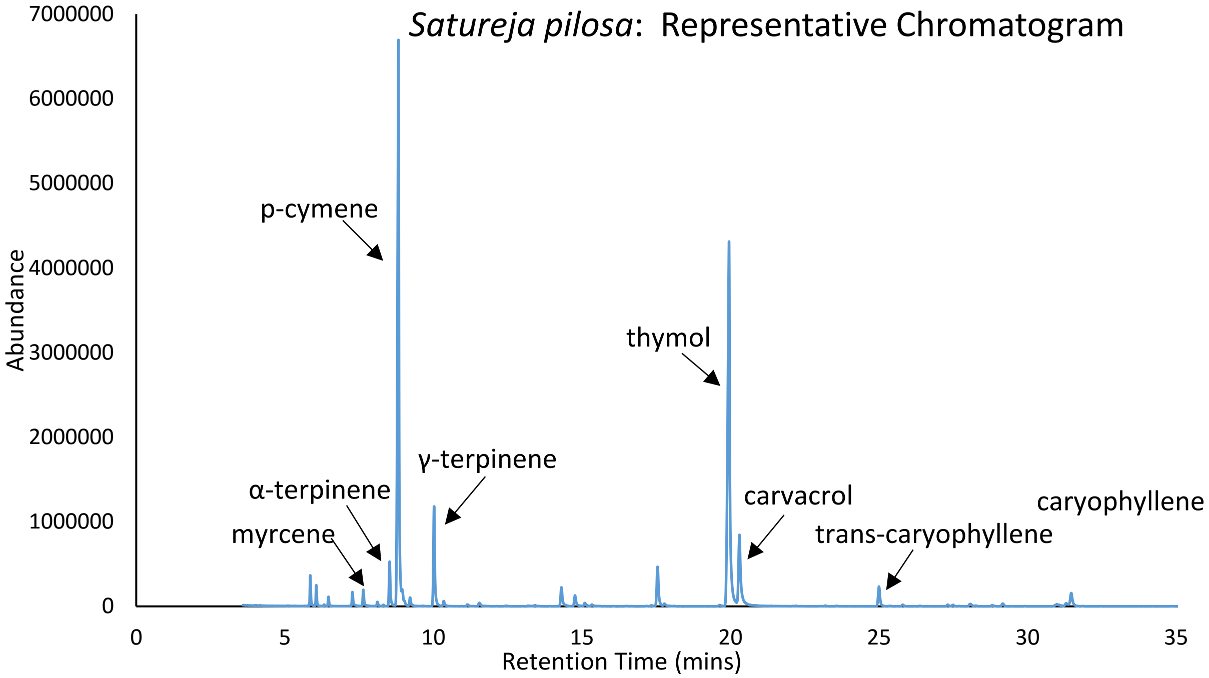
Satureja pilosa: Representative Chromatogram
Abundance
7000000
6000000
5000000
4000000
3000000
2000000
1000000
0
p-cymene
thymol
γ-terpinene
α-terpinene
carvacrol
caryophyllene
myrcene
trans-caryophyllene
0
5
10
15
20
25
30
35
Retention Time (mins)
